# *N* ^1^,*N* ^4^-Diethynyl-*N* ^1^,*N* ^4^-diphenyl­benzene-1,4-diamine

**DOI:** 10.1107/S1600536811045351

**Published:** 2011-11-05

**Authors:** Hideyuki Tabata, Tsunehisa Okuno

**Affiliations:** a Department of Material Science and Chemistry, Wakayama University, Sakaedani, Wakayama 640-8510, Japan

## Abstract

The title compound, C_22_H_16_N_2_, is the first example of an ynamine with H atoms bonded to the terminal C atoms. The environment around each N atom is almost planar. The distances of the N atoms from a least squares plane fitted through each N atom and the surrounding three C atoms, are 0.087 (3) and 0.041 (4) Å. The dihedral angles between these two planes and the central phenyl­ene ring are 23.34 (14) and 34.57 (14)°. The two acetyl­ene groups have an *anti* conformation, keeping a conjugation through the central benzene ring. The freely refined lengths of C_*sp*_—H are 1.00 (5) and 0.93 (4) Å, consistent with those of reported acetyl­enes. The H atoms bound to terminal C atoms have short contacts with the neighboring acetyl­enic C and N atoms. The closest contacts are an H⋯N distance of 2.67 (5) Å and an H⋯C distance of 2.74 (5) Å.

## Related literature

For the related structures of ynamine compounds where a diphenyl­amino group is connected to a diacetyl­ene in the terminal position, see: Galli *et al.* (1988 ▶, 1989 ▶). For the related structures of a diacetyl­ene compound having 9-carbazolyl groups at both ends, see: Mayerle & Flandera (1978 ▶). For the related structures of ynamine compounds incorporating a phenothia­zine-10-yl group, see: Okuno *et al.* (2006 ▶). For our work on the preparation and the structure of the related ynamine mol­ecule incoporating a part of the title compound, see: Tabata *et al.* (2011 ▶).
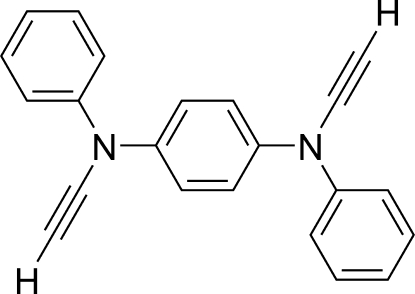


## Experimental

### 

#### Crystal data

C_22_H_16_N_2_
*M* *_r_* = 308.38
Monoclinic, 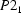

*a* = 9.228 (3) Å
*b* = 7.752 (2) Å
*c* = 11.359 (4) Å
β = 100.880 (4)°
*V* = 798.0 (5) Å^3^
*Z* = 2
Mo *K*α radiation
μ = 0.08 mm^−1^
*T* = 93 K
0.25 × 0.12 × 0.08 mm

#### Data collection

Rigaku Saturn724 diffractometer
Absorption correction: numerical (*NUMABS*; Rigaku, 1999 ▶) *T*_min_ = 0.984, *T*_max_ = 0.994
6457 measured reflections
1942 independent reflections
1627 reflections with *F* ^2^ > 2σ(*F* ^2^)
*R*_int_ = 0.040

#### Refinement

*R*[*F* ^2^ > 2σ(*F* ^2^)] = 0.043
*wR*(*F* ^2^) = 0.093
*S* = 1.03
1939 reflections
282 parameters
1 restraint
All H-atom parameters refined
Δρ_max_ = 0.32 e Å^−3^
Δρ_min_ = −0.32 e Å^−3^

### 

Data collection: *CrystalClear* (Rigaku, 2008 ▶); cell refinement: *CrystalClear*; data reduction: *CrystalClear*; program(s) used to solve structure: *SIR92* (Altomare *et al.*, 1994 ▶); program(s) used to refine structure: *SHELXL97* (Sheldrick, 2008 ▶); molecular graphics: *ORTEP-3* (Farrugia, 1997 ▶); software used to prepare material for publication: *CrystalStructure* (Rigaku, 2010 ▶).

## Supplementary Material

Crystal structure: contains datablock(s) global, I. DOI: 10.1107/S1600536811045351/nk2117sup1.cif

Structure factors: contains datablock(s) I. DOI: 10.1107/S1600536811045351/nk2117Isup2.hkl

Supplementary material file. DOI: 10.1107/S1600536811045351/nk2117Isup3.cml

Additional supplementary materials:  crystallographic information; 3D view; checkCIF report

## Figures and Tables

**Table 1:** The CH⋯π interactions of C_*sp*_—H with the acetylenic carbon and the nitrogen atoms (Å, °)

*D*—H⋯*A*	*D*—H	H⋯*A*	*D*⋯*A*	*D*—H⋯*A*
C20—H1⋯N1^i^	1.00 (5)	2.67 (5)	3.540 (5)	145 (4)
C22—H2⋯C20^ii^	0.93 (4)	2.74 (5)	3.478 (6)	137 (4)

Symmetry codes: (i) 
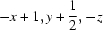
; (ii) 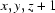.

**Fig. 1.**
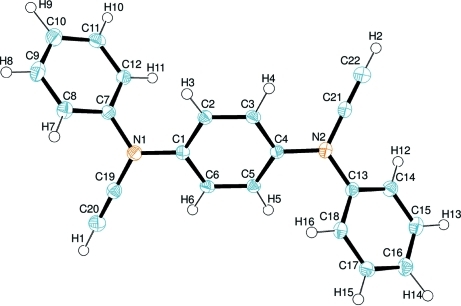
The asymmetric unit of the title compound with atom-numbering scheme. Displacement ellipsoids are drawn at the 50% probability level and H atoms are shown as small spheres.

**Fig. 2.**
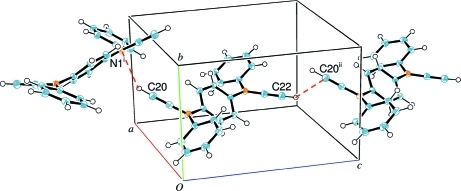
C–H···π interactions between H-atoms of the C-terminals and the acetylenic carbon and the nitrogen atoms. [Symmetry codes: (i) -x + 1, y + 1/2, -z; (ii) x, y, z + 1]

## supplementary crystallographic information

### Comment

Ynamines, where amino groups are connected to acetylene groups, are known to be unstable because of their high reactivity. Therefore, reports of crystal structures are limited to rather stable ynamines (Galli *et al.*, 1988; Galli *et al.*, 1989; Mayerle & Flandera, 1978; Okuno *et al.*, 2006;) which carry some substituents, except for H atoms, on all C– and N-terminals. When H atoms are connected to C– or N-terminals, the stability of ynamines decreases drastically. In the course of our research in ynamine compounds to develop a conjugated linker, (Tabata *et al.*, 2011) we have succeeded in preparation and characterization of the title compound. It is the first example of ynamines with H atoms in the C-terminals.

In the molecular structure, (Fig. 1) the geometric parameters are consistent with those of other reported ynamines. (Table 1) The bond lengths of C*_sp_*—H are 1.00 (5) Å and 0.93 (4) Å, where a marked difference is not recognized compared with those of other acetylenic compounds. The structures around the nitrogen atoms, plane (N1/C1/C7/C19) and plane (N2/C4/C13/C21), are almost planar, where the distances of the nitrogen atoms from the least squares planes are 0.087 (3) Å and 0.041 (4) Å, respectively. The dihedral angles of the plane (N1/C1/C7/C19) with the phenylene (C1/C2/C3/C4/C5/C6) and the phenyl rings (C7/C8/C9/C10/C11/C12) are 23.34 (14)° and 48.74 (15)°, respectively. The dihedral angles of the plane (N2/C4/C13/C21) with the phenylene (C1/C2/C3/C4/C5/C6) and the phenyl rings (C13/C14/C15/C16/C17/C18) are 34.57 (14)° and 29.28 (14)°, respectively. The two acetylene groups have an *anti* conformation, keeping a conjugation through the phenylene ring.

The H-atoms bound to C-terminals have short contacts with the neighboring acetylenic carbon and nitrogen atoms, giving C*_sp_*—H···π interactions. (Fig. 2, Table 2) The C20—H1 bond points to the C2(-*x* + 1, *y* + 1/2, -*z*) atom, where the closest contact is C20—H1···N1(-*x* + 1, *y* + 1/2, -*z*) of 2.67 (5) Å, indicating the interaction with the lone pair of N1. The H2 atom interacts with *p*-orbitals of an adjacent acetylenic carbon atom. The C22—H2 bond directs to the N1(*x*, *y*, *z* + 1) atom, with close contact C22—H2···C20(*x*, *y*, *z* + 1) of 2.74 (5) Å.

### Experimental

*N*^1^,*N*^4^-diethynyl-*N*^1^,*N*^4^-diphenyl-1,4- phenylenediamine

*n*-BuLi in *n*-hexane (15.5 ml, 24.6 mmol) was added dropwise to a solution of *N*^1^,*N*^4^-bis(trichloroethenyl)-*N*^1^, *N*^4^-diphenyl-1,4-phenylenediamine (2.00 g, 3.85 mmol) in dry THF (100 ml) at -78 °C under an argon atmosphere. After the solution was stirred for 1 h, methanol (1.3 ml) was added to the solution. It was allowed to warm to -10 °C and poured into water (50 ml). The water layer was extracted with ether (100 ml), and the combined organic layer was washed with saturated brine (20 ml), and dried over anhydrous sodium sulfate. After the solvent was evaporated, the residue was purified by GPC to give 0.60 g (yield 50%) of the title compound as a reddish brown powder. The single crystals with sufficient quality were obtained by slow evaporation from a solution of chloroform in a refrigerator.

### Refinement

Friedel pairs were merged because the molecule itself was achiral and because there were not any anomalous scattering effects. Four reflections whose 2*θ* angles were lower than 8° were not used for refinement because of the effect of a beam stop. The C-bound H atoms were obtained from a difference Fourier map and were refined isotropically with the restriction of C*_sp_*—H range between 0.95 (4) Å and 1.15 (4) Å.

### Crystal data

**Table:**

C_22_H_16_N_2_	*F*(000) = 324.00
*M**_r_* = 308.38	*D*_x_ = 1.283 Mg m^−^^3^
Monoclinic, *P*2_1_	Mo *K*α radiation, λ = 0.71075 Å
Hall symbol: P 2yb	Cell parameters from 2741 reflections
*a* = 9.228 (3) Å	θ = 3.2–27.5°
*b* = 7.752 (2) Å	µ = 0.08 mm^−^^1^
*c* = 11.359 (4) Å	*T* = 93 K
β = 100.880 (4)°	Prism, colourless
*V* = 798.0 (5) Å^3^	0.25 × 0.12 × 0.08 mm
*Z* = 2	

### Data collection

**Table:**

Rigaku Saturn724 diffractometer	1627 reflections with *F*^2^ > 2σ(*F*^2^)
Detector resolution: 28.445 pixels mm^-1^	*R*_int_ = 0.040
ω scans	θ_max_ = 27.5°
Absorption correction: numerical (*NUMABS*; Rigaku, 1999)	*h* = −11→11
*T*_min_ = 0.984, *T*_max_ = 0.994	*k* = −10→10
6457 measured reflections	*l* = −14→10
1942 independent reflections	

### Refinement

**Table:**

Refinement on *F*^2^	Hydrogen site location: inferred from neighbouring sites
*R*[*F*^2^ > 2σ(*F*^2^)] = 0.043	All H-atom parameters refined
*wR*(*F*^2^) = 0.093	*w* = 1/[σ^2^(*F*_o_^2^) + (0.010*P*)^2^ + 0.5*P*] where *P* = (*F*_o_^2^ + 2*F*_c_^2^)/3
*S* = 1.03	(Δ/σ)_max_ < 0.001
1939 reflections	Δρ_max_ = 0.32 e Å^−^^3^
282 parameters	Δρ_min_ = −0.32 e Å^−^^3^
1 restraint	Extinction correction: *SHELXL97* (Sheldrick, 2008)
Primary atom site location: structure-invariant direct methods	Extinction coefficient: 0.116 (10)
Secondary atom site location: difference Fourier map	

### Fractional atomic coordinates and isotropic or equivalent isotropic displacement parameters (Å^2^)

**Table:**

	*x*	*y*	*z*	*U*_iso_*/*U*_eq_	
N1	0.3719 (3)	0.3729 (3)	0.1482 (2)	0.0205 (6)	
N2	0.7581 (3)	0.3249 (4)	0.5970 (2)	0.0199 (6)	
C1	0.4697 (3)	0.3595 (4)	0.2614 (3)	0.0179 (7)	
C2	0.4381 (3)	0.2478 (4)	0.3489 (3)	0.0185 (7)	
C3	0.5345 (3)	0.2341 (4)	0.4589 (3)	0.0189 (7)	
C4	0.6630 (3)	0.3317 (4)	0.4816 (3)	0.0169 (7)	
C5	0.6951 (3)	0.4417 (4)	0.3933 (3)	0.0178 (7)	
C6	0.5999 (3)	0.4552 (4)	0.2837 (3)	0.0190 (7)	
C7	0.2146 (3)	0.3531 (4)	0.1369 (3)	0.0193 (7)	
C8	0.1366 (4)	0.2700 (4)	0.0355 (3)	0.0240 (8)	
C9	−0.0163 (4)	0.2602 (5)	0.0191 (3)	0.0291 (9)	
C10	−0.0907 (4)	0.3317 (5)	0.1017 (3)	0.0286 (9)	
C11	−0.0121 (4)	0.4117 (4)	0.2036 (3)	0.0249 (8)	
C12	0.1407 (3)	0.4234 (4)	0.2211 (3)	0.0208 (7)	
C13	0.9137 (3)	0.3538 (4)	0.6126 (3)	0.0194 (7)	
C14	0.9879 (3)	0.4337 (4)	0.7169 (3)	0.0226 (8)	
C15	1.1393 (4)	0.4586 (4)	0.7329 (3)	0.0263 (8)	
C16	1.2172 (4)	0.4105 (4)	0.6452 (3)	0.0262 (8)	
C17	1.1418 (3)	0.3325 (4)	0.5412 (3)	0.0229 (8)	
C18	0.9911 (3)	0.3013 (4)	0.5250 (3)	0.0212 (7)	
C19	0.4187 (3)	0.4520 (4)	0.0562 (3)	0.0223 (7)	
C20	0.4608 (4)	0.5242 (4)	−0.0232 (3)	0.0266 (9)	
C21	0.6955 (3)	0.3114 (4)	0.6945 (3)	0.0208 (7)	
C22	0.6419 (4)	0.3023 (4)	0.7811 (3)	0.0258 (8)	
H1	0.498 (5)	0.586 (6)	−0.089 (4)	0.056 (13)*	
H2	0.601 (4)	0.299 (6)	0.850 (3)	0.039 (10)*	
H3	0.352 (4)	0.178 (5)	0.330 (3)	0.017 (8)*	
H4	0.502 (4)	0.162 (5)	0.523 (3)	0.024 (9)*	
H5	0.785 (4)	0.509 (5)	0.404 (3)	0.021 (8)*	
H6	0.624 (4)	0.529 (5)	0.223 (3)	0.033 (10)*	
H7	0.194 (3)	0.217 (4)	−0.021 (3)	0.014 (7)*	
H8	−0.064 (4)	0.207 (5)	−0.054 (3)	0.025 (9)*	
H9	−0.218 (4)	0.329 (6)	0.084 (3)	0.038 (10)*	
H10	−0.065 (4)	0.464 (5)	0.262 (3)	0.028 (9)*	
H11	0.191 (4)	0.486 (5)	0.294 (3)	0.033 (10)*	
H12	0.934 (4)	0.468 (5)	0.779 (3)	0.028 (9)*	
H13	1.195 (4)	0.509 (5)	0.811 (3)	0.033 (10)*	
H14	1.336 (4)	0.424 (6)	0.662 (3)	0.038 (10)*	
H15	1.196 (4)	0.298 (5)	0.478 (3)	0.025 (8)*	
H16	0.939 (4)	0.242 (5)	0.449 (3)	0.028 (9)*	

### Atomic displacement parameters (Å^2^)

**Table:**

	*U*^11^	*U*^22^	*U*^33^	*U*^12^	*U*^13^	*U*^23^
N1	0.0205 (12)	0.0205 (12)	0.0205 (12)	−0.0003 (10)	0.0036 (10)	0.0013 (10)
N2	0.0194 (11)	0.0194 (12)	0.0194 (12)	0.0000 (10)	0.0000 (9)	0.0000 (10)
C1	0.0179 (13)	0.0179 (14)	0.0179 (13)	0.0027 (11)	0.0034 (11)	−0.0016 (11)
C2	0.0186 (14)	0.0186 (14)	0.0186 (14)	−0.0024 (11)	0.0039 (11)	−0.0004 (11)
C3	0.0192 (14)	0.0192 (13)	0.0192 (14)	0.0003 (11)	0.0056 (11)	0.0029 (11)
C4	0.0170 (13)	0.0170 (13)	0.0170 (13)	0.0017 (11)	0.0040 (10)	−0.0007 (12)
C5	0.0179 (14)	0.0179 (13)	0.0179 (13)	−0.0012 (12)	0.0039 (11)	−0.0003 (11)
C6	0.0194 (14)	0.0194 (14)	0.0194 (13)	−0.0005 (12)	0.0070 (11)	0.0025 (12)
C7	0.0192 (14)	0.0192 (14)	0.0192 (14)	−0.0002 (12)	0.0031 (11)	0.0032 (11)
C8	0.0238 (15)	0.0238 (15)	0.0238 (15)	0.0005 (12)	0.0027 (13)	−0.0014 (12)
C9	0.0278 (16)	0.0278 (17)	0.0278 (17)	−0.0046 (13)	−0.0046 (14)	0.0009 (13)
C10	0.0280 (17)	0.0280 (16)	0.0280 (17)	−0.0030 (14)	0.0005 (13)	0.0080 (15)
C11	0.0254 (16)	0.0254 (15)	0.0254 (15)	0.0012 (13)	0.0088 (13)	0.0054 (13)
C12	0.0207 (14)	0.0207 (14)	0.0207 (14)	−0.0018 (12)	0.0030 (12)	0.0017 (12)
C13	0.0190 (14)	0.0190 (14)	0.0190 (14)	−0.0004 (12)	0.0009 (11)	0.0029 (11)
C14	0.0225 (15)	0.0225 (14)	0.0225 (15)	−0.0008 (13)	0.0034 (12)	−0.0011 (13)
C15	0.0253 (16)	0.0253 (16)	0.0253 (16)	−0.0015 (13)	−0.0031 (13)	0.0007 (13)
C16	0.0256 (16)	0.0256 (16)	0.0256 (16)	0.0004 (13)	0.0000 (13)	0.0055 (13)
C17	0.0232 (15)	0.0232 (15)	0.0232 (14)	0.0068 (13)	0.0068 (12)	0.0067 (13)
C18	0.0208 (14)	0.0208 (14)	0.0208 (14)	0.0021 (12)	0.0005 (12)	0.0015 (12)
C19	0.0221 (14)	0.0221 (14)	0.0221 (14)	−0.0003 (12)	0.0028 (11)	−0.0043 (12)
C20	0.0267 (17)	0.0267 (16)	0.0267 (16)	−0.0017 (13)	0.0057 (13)	0.0010 (13)
C21	0.0203 (14)	0.0203 (14)	0.0203 (14)	0.0000 (12)	0.0000 (11)	0.0000 (12)
C22	0.0260 (15)	0.0260 (16)	0.0260 (15)	−0.0004 (13)	0.0067 (13)	0.0003 (13)

### Geometric parameters (Å, °)

**Table:**

N1—C1	1.428 (4)	C15—C16	1.385 (6)
N1—C7	1.441 (4)	C16—C17	1.391 (5)
N1—C19	1.351 (5)	C17—C18	1.389 (4)
N2—C4	1.434 (4)	C19—C20	1.187 (5)
N2—C13	1.431 (4)	C21—C22	1.184 (6)
N2—C21	1.346 (5)	C2—H3	0.95 (4)
C1—C2	1.390 (5)	C3—H4	1.01 (4)
C1—C6	1.394 (4)	C5—H5	0.97 (4)
C2—C3	1.394 (5)	C6—H6	0.95 (4)
C3—C4	1.389 (4)	C8—H7	0.99 (4)
C4—C5	1.390 (5)	C9—H8	0.96 (4)
C5—C6	1.386 (5)	C10—H9	1.15 (4)
C7—C8	1.395 (5)	C11—H10	0.98 (4)
C7—C12	1.387 (5)	C12—H11	1.00 (4)
C8—C9	1.390 (6)	C14—H12	0.97 (4)
C9—C10	1.379 (6)	C15—H13	1.02 (4)
C10—C11	1.391 (5)	C16—H14	1.08 (4)
C11—C12	1.389 (5)	C17—H15	0.99 (4)
C13—C14	1.396 (5)	C18—H16	1.02 (4)
C13—C18	1.391 (5)	C20—H1	1.00 (5)
C14—C15	1.388 (5)	C22—H2	0.93 (4)
			
C1—N1—C7	121.8 (3)	N1—C19—C20	178.7 (4)
C1—N1—C19	119.3 (3)	N2—C21—C22	178.7 (4)
C7—N1—C19	116.3 (3)	C1—C2—H3	118 (2)
C4—N2—C13	122.3 (3)	C3—C2—H3	122 (2)
C4—N2—C21	118.1 (3)	C2—C3—H4	117.6 (19)
C13—N2—C21	119.1 (3)	C4—C3—H4	121.9 (19)
N1—C1—C2	120.4 (3)	C4—C5—H5	122 (2)
N1—C1—C6	120.1 (3)	C6—C5—H5	117 (2)
C2—C1—C6	119.4 (3)	C1—C6—H6	120 (2)
C1—C2—C3	120.2 (3)	C5—C6—H6	120 (2)
C2—C3—C4	120.2 (3)	C7—C8—H7	118.0 (16)
N2—C4—C3	120.2 (3)	C9—C8—H7	122.8 (16)
N2—C4—C5	120.3 (3)	C8—C9—H8	115 (3)
C3—C4—C5	119.4 (3)	C10—C9—H8	124 (3)
C4—C5—C6	120.6 (3)	C9—C10—H9	119.7 (19)
C1—C6—C5	120.1 (3)	C11—C10—H9	120 (2)
N1—C7—C8	118.5 (3)	C10—C11—H10	120 (2)
N1—C7—C12	121.0 (3)	C12—C11—H10	120 (2)
C8—C7—C12	120.4 (3)	C7—C12—H11	124 (3)
C7—C8—C9	119.2 (4)	C11—C12—H11	117 (3)
C8—C9—C10	120.7 (3)	C13—C14—H12	120 (2)
C9—C10—C11	119.8 (4)	C15—C14—H12	120 (2)
C10—C11—C12	120.3 (4)	C14—C15—H13	120 (3)
C7—C12—C11	119.6 (3)	C16—C15—H13	119 (3)
N2—C13—C14	119.6 (3)	C15—C16—H14	120 (2)
N2—C13—C18	120.3 (3)	C17—C16—H14	121 (2)
C14—C13—C18	120.1 (3)	C16—C17—H15	120 (2)
C13—C14—C15	119.5 (3)	C18—C17—H15	119 (2)
C14—C15—C16	121.0 (3)	C13—C18—H16	121 (3)
C15—C16—C17	118.9 (4)	C17—C18—H16	120 (3)
C16—C17—C18	121.1 (4)	C19—C20—H1	179 (3)
C13—C18—C17	119.4 (3)	C21—C22—H2	178 (3)

### Hydrogen-bond geometry (Å, °)

**Table:**

*D*—H···*A*	*D*—H	H···*A*	*D*···*A*	*D*—H···*A*
C20—H1···N1^i^	1.00 (5)	2.67 (5)	3.540 (5)	145 (4)
C22—H2···C20^ii^	0.93 (4)	2.74 (5)	3.478 (6)	137 (4)

Symmetry codes: (i) −*x*+1, *y*+1/2, −*z*; (ii) *x*, *y*, *z*+1.
